# Environmental problems of emerging toxic metals and treatment technology and methods

**DOI:** 10.1039/d4ra06085g

**Published:** 2024-11-25

**Authors:** Yanhao Zhang, Zhiyuan Shen, Wenlu Zhou, Chengying Liu, Yi Li, Botao Ding, Peng Zhang, Xu Zhang, Zhibin Zhang

**Affiliations:** a School of Municipal and Environmental Engineering, Shandong Jianzhu University Jinan 250101 China 11363@sdjzu.edu.cn; b Shandong Academy for Environmental Planning Jinan 250101 China; c Yantai Economic and Technological Development Zone Water Supply Co., Ltd Yantai 264006 China; d Research Center for Eco-Environmental Sciences, Chinese Academy of Sciences Beijing 100085 China

## Abstract

The increasing industrial use of toxic metals essential for modern electronics and renewable energy presents significant environmental and health challenges. This review was needed to address the environmental risks posed by toxic metals, particularly those accumulating in soil and sediment ecosystems. The objective is to examine the sources of toxic metal pollution, their ecological impacts, and the effectiveness of existing treatment technologies. By comprehensively reviewing the recent literature, we analyzed the physiological and molecular responses of plants to toxic metals, focusing on their toxicity mechanisms. Key parameters measured include toxic metal concentration, soil and sediment health, microbial diversity, and plant stress responses. Our findings highlight that toxic metals, such as lithium, nickel, and indium, fueled by industrial activities, including mining and electronic waste disposal, significantly disrupt ecosystems. These metals bioaccumulate, harming soil microbial communities and aquatic life. For instance, in soil ecosystems, cadmium and lead inhibit microbial functions, while in aquatic systems, resuspension of sediment-bound metals leads to persistent contamination. Data show that phytoremediation and microbial techniques are effective in reducing toxic metal concentrations up to 30–40%. In conclusion, long-term monitoring and sustainable remediation strategies are essential to mitigate these environmental impacts. Future efforts should focus on enhancing the efficiency of bioremediation techniques and integrating these methods into global toxic metal management practices.

## Abstract 2

The increasing industrial use of toxic metals essential for modern electronics and renewable energy presents significant environmental and health challenges.

## Graphical abstract

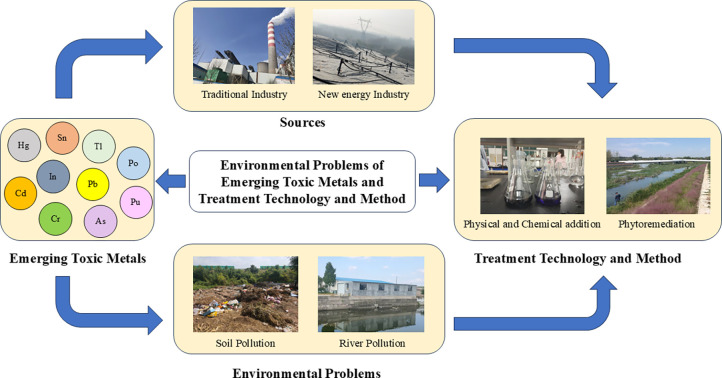

## 1 Introduction

Electronic pollution is mainly caused by waste and expired electronic products.^1^ The waste contains many non-ferrous metals, ferrous metals, plastics, rubber, glass and other recyclable renewable resources.^2^ Some waste electronic products also contain emerging toxic metals such as lithium, nickel and indium. Electronic products contain a large number of harmful and toxic substances; if discarded and buried at will, they will produce large amounts of liquid and residue wastes, causing serious environmental pollution.^3–6^ Toxic metals, pivotal in the development of modern electronics and renewable energy technologies, have seen a surge in demand across various industrial sectors.^7^ This group of elements, often rare and possessing unique properties, is integral to advancing technology and energy solutions.^8–11^ However, their extraction and utilization have raised significant environmental concerns due to their potential adverse impacts on ecosystems, particularly soil and sediments, where they tend to accumulate and persist.^12–15^

Toxic metals pose significant risks to both environmental and human health owing to their toxicity, persistence, and bioaccumulation.^16^ To provide a clearer understanding of their toxicological impact, we have included LD_50_ (lethal dose 50) values for several key toxic metals, which represent the dose required to cause death in 50% of a test population. For example, the LD_50_ of cadmium (Cd) in rats is approximately 225 mg kg^−1^, highlighting its high toxicity even at low concentrations. Similarly, lead (Pb) has an LD_50_ of around 500 mg kg^−1^ in rats, and chronic exposure can lead to neurotoxicity and damage to vital organs.^17^ Nickel (Ni), with an LD_50_ of 350 mg kg^−1^, is known for its harmful effects on the respiratory system, while indium (In) has an LD_50_ of 1000 mg kg^−1^, and its potential for long-term environmental accumulation remains a concern.

This review delves into the multifaceted issue of toxic metal pollution, focusing on the environmental status of these contaminants within soil and sediment ecosystems. It addresses the critical sources of toxic metals, including mining, industrial processes, and electronic waste disposal, which contribute significantly to environmental loads. The pathways through which these metals enter and distribute within ecosystems are complex and influenced by both natural processes and anthropogenic activities. Furthermore, this review examines the ecological impacts of toxic metals, including disruptions to microbial communities and adverse effects on plant health, which can cascade through food webs affecting aquatic and terrestrial life forms, thereby posing potential risks to human health. The treatment technologies for toxic metal removal from contaminated sites are also explored, highlighting innovative approaches such as phytoremediation and advanced microbial techniques, which offer promising solutions to mitigate these pollutants effectively.

The primary objectives of this review are: to thoroughly investigate the sources and pathways of toxic metal pollution in soil and sediment ecosystems; to examine the environmental fate and distribution of these metals within these critical ecosystems; to analyze the ecological effects of toxic metal pollution, emphasizing the consequent impacts on soil and sediment health and the broader implications for human health; to evaluate both traditional and innovative monitoring and analytical techniques that aid in detecting and quantifying toxic metal contamination; to provide a comprehensive overview of both existing and emerging treatment technologies designed to remove or mitigate toxic metal contamination from the soil and sediment; to discuss the ongoing challenges and propose future directions for research and policy-making in the field of toxic metal pollution mitigation. By addressing these points, this review aims to underscore the importance of a multi-disciplinary approach in tackling toxic metal pollution, emphasizing the need for integrated strategies that protect both environmental health and human welfare.

## 2 Methodology

This review describes recent advancements in the study of toxic metal pollution, its ecological impacts, and treatment technologies through a comprehensive literature review of peer-reviewed articles, reports, and case studies published between 2015 and 2023. Studies were selected based on several key criteria: relevance to the environmental impacts of emerging toxic metals (such as lithium, nickel, and indium), geographic diversity, inclusion of experimental and observational data, and methodological rigor (*e.g.*, studies that employed robust statistical analysis and/or field trials). Databases such as Web of Science, Scopus, and Google Scholar were used to identify the studies, ensuring research coverage from various ecological contexts.

To ensure a balanced and thorough analysis, we included studies from different regions that provided data on the behavior and remediation of toxic metals in both soil and sediment ecosystems. Special emphasis was placed on research investigating the effects of toxic metals on microbial communities, soil health, and aquatic systems, as well as biological and technological approaches to mitigate these impacts.

The studies were analyzed based on the type of toxic metal, the environmental matrix (soil or sediment), and the remediation techniques employed. Quantitative data were extracted from experimental studies to compare the effectiveness of different remediation methods in reducing metal concentrations, restoring ecosystem functions, and enhancing biodiversity. The review categorized the findings into three main areas: sources and pathways of toxic metal pollution, ecological effects, and the efficacy of remediation technologies.

This review not only describes findings across various studies but also compares them based on factors such as cost, sustainability, environmental impact, and long-term effectiveness. By systematically analyzing the data from multiple studies, we provide a comparative analysis of the remediation strategies and their practical applicability in different environmental contexts.

## 3 Source and environmental trend

Toxic metal pollution in soil and sediment ecosystems arises from various sources and pathways.^18–20^ Understanding the source pathways of toxic metals and their environmental fate and distribution is critical to assessing their impact on soil and sediment ecosystems.^21^ This section explores the main causes of toxic metal pollution in these ecosystems and related factors affecting toxic metal behavior in the environment.

### 3.1 Sources and pathways in the soil and sediment

One of the central sources of toxic metal pollution in the soil and sediment originates from mining and ore processing activities.^22^ Toxic metals are often extracted as a by-product of zinc, tin, and other base metal mining operations.^23,24^ During the extraction and processing of these ores, materials rich in toxic metals can be released into the environment, resulting in the contamination of nearby soil and sediments.^25–28^

The manufacturing and processing of toxic metal-containing products, such as semiconductors, thin-film solar cells, and touchscreens, can lead to the emission of toxic metal particles and compounds into the atmosphere.^29^ These emissions contribute to atmospheric deposition, and subsequently, toxic metals can settle in the soil and sediment through deposition from the air.^30^

The disposal and recycling of electronic devices, many of which incorporate toxic metals in the form of toxic tin oxide (ITO) coatings, represent a significant source of toxic metal pollution in the soil and sediments.^31–34^ Improper electronic waste management, including inadequate recycling practices and landfill disposal, poses a substantial risk of releasing toxic metals into the environment.^35^

Agricultural practices also cause soil pollution by using toxic metal-containing fertilizers or pesticides. This raises concerns about the potential accumulation of toxic metals in crops and their entry into the terrestrial food chain.^36^ While agricultural inputs may not be the primary source of toxic metal pollution, they can contribute to localized contamination.^37^

### 3.2 Environmental fate and distribution

Factors such as soil and sediment composition, pH, and organic matter affect toxic metal migration in the soil.^38^ Generally, toxic metals tend to adsorb on soil particles, reducing their mobility.^39^ However, this adsorption can vary depending on local conditions, with more acidic soils potentially experiencing reduced sorption of toxic metals.

The bioavailability of toxic metals in the soil and sediment plays a pivotal role in determining their ecological impact. Specific soil conditions, such as pH and the presence of chelating agents, can influence the uptake of toxic metals by plants.^40,41^ Consequently, toxic metals can enter terrestrial food webs, affecting wildlife and potentially posing risks to human consumers. In aquatic environments, toxic metals can accumulate in sediments over time. These sediment-bound toxic metals can pose a potential risk to aquatic ecosystems. During sediment resuspension events caused by natural processes or human activities, toxic metals can be released back into the water column.^42^ This cycle can perpetuate the contamination of aquatic systems.

## 4 Ecological effects of toxic metals

Toxic metal pollution significantly affects soil and sediment ecosystems, which are crucial for environmental health. This section investigates the ecological impacts of toxic metals, particularly how they disrupt microbial diversity and soil fertility. Metals like cadmium, lead, and mercury can inhibit microbial functions and reduce soil structure stability. Additionally, toxic metals accumulate in sediments, affecting benthic organisms and potentially leading to bioaccumulation up the food chain. This section also considers the broader implications of these disruptions, including impacts on plant growth and food chains and the interactions with other environmental stressors.

Toxic metal pollution significantly affects soil and sediment ecosystems, which are critical to environmental health.^43^ In addition to the direct impacts of toxic metals, interactions with other environmental stressors such as organic pollutants, climate change, and nutrient imbalances can exacerbate the ecological damage. For instance, toxic metals may synergistically interact with persistent organic pollutants, increasing their bioavailability and toxicity to aquatic organisms and plants.^44^ Such combined pollution can lead to more severe disruptions in nutrient cycling and microbial community composition, further degrading soil fertility and ecosystem stability. Bioaccumulation of toxic metals through the food chain, especially in regions affected by multiple stressors, can increase the risk of exposure to toxic metals such as cadmium, mercury, and lead. Communities living near polluted sites or relying on contaminated agricultural or aquatic products are particularly vulnerable.^45^ Long-term exposure to these metals is linked to severe health outcomes, including cardiovascular diseases, neurotoxicity, and cancer. The interaction between toxic metal pollution and other environmental stressors is a complex environmental problem that not only affects environmental quality but also poses a potential threat to human health.^46^ Toxic metals enter the environment mainly through industrial pollution, traffic pollution and household garbage pollution, and then affect the soil, water and air quality.^47^ These pollutants do not exist in isolation in the environment but interact with other environmental stressors, such as chemicals and biotoxins, leading to complex pollution, which has a more extensive and far-reaching impact on the ecosystem.^48^

### 4.1 Soil and aquatic ecosystems

Studies have shown that elevated toxic metal concentrations in soil can adversely affect soil microbial communities, earthworms, and plant growth.^49–51^ Toxic metal interference with nutrient cycling and soil enzyme activity can lead to reduced soil fertility. These effects can disrupt the delicate balance of soil ecosystems, impacting overall soil health and productivity.

Toxic metal contamination in sediments can have significant consequences for aquatic ecosystems. It can impair the health of aquatic organisms, such as fish and benthic invertebrates, with potential implications for food webs and ecosystem dynamics.^52^ Moreover, the resuspension of sediment-bound toxic metals can contribute to the ongoing contamination of aquatic systems.

### 4.2 Microbial community

Microorganisms are important participants in soil remediation of toxic metals, and some sensitive microbial communities respond to toxic metal toxicity, resulting in changes in the community structure and microbial community diversity.^53^ The toxicity of toxic metal pollution to microorganisms will destroy the relevant functions of the ecosystem, such as inhibiting the activity of microbial enzymes related to biological activities such as nitrogen fixation, nitrification and denitrification. This disruption of microbial-mediated nutrient conversion can have knock-on effects on ecosystem productivity and nutrient availability.^1,54^ In addition, stress responses based on the resistance of microorganisms to toxic metal-induced stress may produce associated proteins and metabolites, resulting in changes in the metabolism and energy distribution of microorganisms, impinging on their ability to perform essential ecosystem functions.^55^ The effects of toxic metals can also extend to higher nutrient levels, affecting the health of organisms that rely on microbially driven processes.^56^ The toxic metal-contaminated soil will have different effects on the growth and development of soil microorganisms and plants and enter the human body through the food chain. In summary, toxic metal contamination has a multifaceted impact on the sediment and soil microbiota, affecting microbial community composition, diversity, and function. Understanding these impacts is critical to assessing the ecological risks associated with toxic metal pollution and developing effective remediation strategies to mitigate its adverse effects on ecosystem health.

### 4.3 Phytotoxic effect

While *Arabidopsis thaliana* offers invaluable insights into the phytotoxicity of toxic metals, examining its effects across a wider range of species, including crops and native plants, is paramount.^57^ This broader analysis reveals varying degrees of toxic metal tolerance and accumulation patterns, highlighting the ecological and food security implications of toxic metal contamination. The elucidation of stress response pathways in plants reveals a sophisticated network of signaling mechanisms activated by toxic metal exposure.^58^ From the induction of antioxidant enzymes to the modulation of hormone signaling pathways, plants exhibit a dynamic response to mitigate toxic metal-induced stress, underscoring the resilience and adaptability of the plant kingdom. Understanding the cellular and molecular underpinnings of toxic metal toxicity is crucial for developing effective countermeasures.^59^ Toxic metals interact with cellular membranes, proteins, and deoxyribonucleic acid,^42^ leading to oxidative stress, metabolic disruption, and impaired growth and development. The identification of specific genes and proteins targeted by toxic metals offers potential biomarkers for monitoring environmental exposure and toxicity.

### 4.4 Human health concerns

While environmental impact is the focus of this review, it is essential to note that the threat of toxic metal pollution to human health cannot be ignored. The potential transfer of toxic metals through the food chain, particularly from crops grown in contaminated soil or from the consumption of contaminated aquatic organisms, poses a potential risk to human consumers.^60^ However, further research is needed to fully assess the health risks associated with toxic metal contamination. Toxic metal exposure has significant effects on human health and could lead to cardiovascular disease, nerve damage, kidney damage, diabetes and even cancer. In particular, exposure to toxic metals such as lead, cadmium, mercury and arsenic is particularly harmful to human health.^61^ The elderly and children are the most vulnerable groups in society, and their exposure to toxic metal pollution will vary due to differences in physiological characteristics, different living habits and external environmental intervention. For example, the elderly may ingest toxic metals primarily through food near mining sites, while children may be exposed to toxic metals through air inhalation and soil contact.^62^ The accumulation of toxic metals in the human body, to a certain extent, will cause chronic poisoning, affect the activity of proteins and enzymes, and lead to various health problems. For example, overall mortality and malignant tumor mortality were significantly higher in the high-exposure group than in the low-exposure group.^63^ In addition, toxic metals may also cause prenatal defects, respiratory diseases, heart diseases and mental disorders. Exposure to toxic metals can be reduced and excretion can be increased through diet, nutrition and lifestyle choices.

## 5 Evaluation and management technology analysis

### 5.1 Analytical techniques for detection and evaluation of toxic metals

Efficient detection and monitoring of toxic metal contamination are crucial for addressing this environmental issue. This section discusses various analytical techniques and risk assessment models used for the detection and assessment of toxic metals. Several analytical methods are employed to detect and quantify toxic metals in soil and sediment samples. These methods include spectroscopic techniques, mass spectrometry, and chemical analysis. Technical methods have two objectives, *i.e.*, to consider their advantages and disadvantages, as well as applicable conditions, such as sample type, required sensitivity and cost considerations.^64^

Risk assessment models are essential tools for evaluating the potential ecological and human health risks associated with toxic metal pollution.^65^ These models consider factors such as exposure pathways, toxicity data, and environmental concentrations to estimate the risk levels posed by toxic metal contamination. Proper risk assessment is critical for making informed decisions about remediation efforts and regulatory actions.

### 5.2 Treatment technologies for emerging toxic metals

Addressing emerging toxic metal pollution in the soil and sediment requires effective treatment technologies. This section explores various treatment methods used for the removal of emerging toxic metals from contaminated environments.

Chemical precipitation involves the addition of specific chemicals to contaminated water or soil to induce the formation of insoluble toxic metal compounds that can be easily separated and removed.^66^ This method is effective for reducing toxic metal concentrations in wastewater and contaminated soil. Adsorption techniques use materials, such as activated carbon, zeolites, or other sorbents, to adsorb toxic metal ions from solutions. These materials have high surface areas and can selectively bind to toxic metals, effectively removing them from water or soil matrices.^67^

In order to solve the adverse problem of toxic metal pollution on the environment, the use of ubiquitous microorganisms for remediation is undoubtedly a good choice.^68^ The relevant microbial ecosystem is constructed in toxic metal-contaminated soil, and its metabolic capacity is used to degrade or fix toxic metal pollutants in the soil or water environment to achieve the remediation of soil-polluting toxic metals and achieve sustainable development.^11^ For example, bioremediation methods and bacteria or fungi that can metabolize toxic metal-related compounds to reduce the harm of toxic metal pollutants can be incorporated into biomass.^69^ Another example is the use of phytoremediation supplemented by microorganisms in the relevant contaminated areas to find specific microorganisms; through the symbiotic relationship, microorganisms promote the ability of plants to absorb and chelate toxic metals from soil or water, thus enhancing the toxic metal accumulation of plants to absorb and detoxify toxic metals.^70^ In addition, newly developing technologies, such as genetic engineering, can also be used to develop genetically modified microorganisms with enhanced toxic metal repair capabilities.^71^ In order to achieve the purpose of efficient removal of toxic metals, the researchers manipulated the microbial genes involved in toxic metal metabolism to develop strains. In general, these methods are cost-effective and environmentally friendly for the remediation of toxic metal-contaminated sites, and have considerable application prospects. Microbial remediation technology provides sustainable and environmentally friendly solutions to protect the environment and public health. Continued research and innovation in this field has the potential to further improve the efficiency and applicability of these methods in the fight against toxic metal pollution on a global scale.

There are many methods of phytoremediation, mainly planting plants in the soil or sediment to achieve the purpose of extracting, stabilizing or degrading toxic metals. Certain plant species have the ability to accumulate toxic metals in their tissues, which can then be harvested and disposed of properly.^72^ Phytoremediation is an environmentally friendly approach to toxic metal cleanup. Leveraging genetic engineering and synthetic biology to design microbes with heightened metal tolerance and bioremediation capabilities represents a frontier in environmental biotechnology.

### 5.3 Cost-effectiveness, sustainability and environmental impact comparison

When evaluating treatment technologies for toxic metal pollution, it is essential to consider three key factors: cost-effectiveness, long-term sustainability, and environmental impact. These criteria determine not only the immediate effectiveness of the treatment but also the feasibility of long-term applications and their ecological consequences.

Chemical precipitation is a widely used method for the removal of toxic metals, known for its high efficiency in reducing contaminant concentrations in water and soil. However, this method is costly, and a high initial capital investment is required for the chemicals and equipment.^73^ Additionally, the long-term environmental risks associated with chemical precipitation are notable since toxic metals may remain in the treated environment, bound in insoluble forms. Continuous use of chemicals can also lead to secondary pollution, reducing the sustainability of this approach. In large-scale remediation efforts, the high costs and potential ecological risks make this method suitable only for urgent or high-priority contamination scenarios where quick results are needed.

Adsorption technologies, including the use of materials like activated carbon and other sorbents, offer a more environmentally friendly and cost-effective alternative to chemical methods. These materials can selectively bind to toxic metal ions, effectively removing them from contaminated matrices. However, the regeneration and disposal of spent adsorbents can add significant costs to the process. Moreover, in large contaminated areas, scalability becomes a limiting factor, as the capacity of adsorbents to remove pollutants is finite, and replacing or regenerating these materials is costly and resource-intensive. Despite these challenges, adsorption technologies are valued for their lower environmental impact compared to chemical methods.^74^

Microbial remediation leverages naturally occurring or engineered microorganisms to degrade or stabilize toxic metals in the environment. This method is relatively low-cost and has a high degree of environmental compatibility, as it promotes the natural degradation of pollutants. Microbial remediation is highly adaptable to different environmental conditions, but it requires specific environmental factors, such as optimal temperature and nutrient availability, to perform effectively.^75^ Additionally, microbial processes are slower than chemical methods, which can be a drawback when rapid remediation is required. However, in terms of long-term sustainability, microbial remediation is superior, as it avoids secondary pollution and promotes ecological restoration.

Phytoremediation involves the use of plants to absorb, stabilize, or degrade toxic metals from soil and sediments. This approach is cost-effective, particularly in large-scale applications, as it requires minimal inputs once the plants are established. Phytoremediation is also environmentally sustainable, as it enhances the ecological balance by promoting plant growth and biodiversity. However, the main limitation of phytoremediation is its slow rate of contaminant removal, and the harvested plant material must be disposed of carefully to prevent secondary contamination. Despite these challenges, phytoremediation remains a viable option for long-term remediation projects, especially in large, low-to-moderate contaminated areas (Table 1).

**Table 1:** Advantages and disadvantages of different remediation methods

Remediation method	Cost	Scalability	Sustainability	Long-term effectiveness	Ecological impact
Chemical precipitation	High initial investment; highly efficient	Highly scalable, suitable for large areas by adding chemicals	Low sustainability due to continuous chemical use and potential for secondary pollution	Rapid contaminant reduction but may require repeated treatments for desired outcomes	Negative ecological impact, can lead to chemical residues in soil and water systems, potentially harming ecosystems long-term
Adsorption technologies	Moderate cost; high regeneration and disposal costs	Limited scalability, especially in large contaminated areas	More environmentally friendly but requires resources for adsorbent regeneration	Effective in the short-term but long-term costs and disposal challenges reduce sustainability	Moderate ecological impact; relatively safe for ecosystems, but improper disposal of spent adsorbents can cause localized contamination
Microbial remediation	Low initial cost but slower process	Adaptable to various environments but requires specific conditions for optimal performance	High sustainability with minimal environmental disruption	Stable results but long processing time depending on environmental factors	Positive ecological impact; enhances biodiversity and soil health using naturally occurring or engineered microbes to degrade contaminants
Phytoremediation	Low cost; minimal inputs required after initial setup	Suitable for large-scale pollution areas	Environmentally friendly and enhances ecological sustainability	Slow process with potential for secondary contamination through harvested plant disposal	Positive ecological impact; improves ecosystem functions and biodiversity, but disposal of contaminated plant material must be managed to prevent recontamination

#### 5.3.1 Cost-effectiveness

Chemical precipitation provides immediate results but comes with significant financial costs in terms of initial investment and long-term operational expenses related to chemical procurement. In contrast, microbial and phytoremediation methods offer much lower initial costs, but the time required for remediation may lengthen the overall cost of managing a contaminated site. Adsorption technologies fall in the middle, being less costly upfront but incurring high costs for adsorbent regeneration and disposal.

#### 5.3.2 Sustainability

Microbial and phytoremediation methods are generally considered more sustainable over the long term. These methods rely on biological processes to degrade or stabilize contaminants, minimizing the need for external inputs and avoiding the generation of secondary pollutants. Chemical precipitation, while highly effective, lacks sustainability due to its dependence on continuous chemical applications, which may harm the environment over time. Adsorption technologies, though greener than chemical methods, still require careful management to handle spent adsorbents responsibly.

#### 5.3.3 Environmental impact

Phytoremediation and microbial remediation stand out for their minimal environmental footprint. These approaches use natural processes that restore ecological balance, making them ideal for long-term remediation with limited environmental disruption. Chemical methods, though efficient, may cause lasting damage to soil and water systems through chemical residues and secondary pollution. Adsorption methods are more benign but pose the risk of pollution if spent adsorbents are not handled properly.

### 5.4 Environmental effects to treatment strategies

A comprehensive understanding of the environmental effects of toxic metal contamination is critical in developing effective treatment strategies. The ecological impacts of toxic metals, such as their bioaccumulation in food chains, toxicity to microbial communities, and disruption of nutrient cycles, inform the selection of appropriate remediation techniques.^76^ For example, metals like cadmium and lead, which are highly toxic to microbial communities, may benefit from microbial remediation methods tailored to restore microbial diversity and functions. On the other hand, metals such as indium, which persist in sediments and disrupt aquatic ecosystems, may require sediment-based adsorption or phytoremediation approaches that focus on long-term metal immobilization and removal.^77^ By identifying the specific ecological impacts of each toxic metal, researchers can tailor remediation efforts to address the particular environmental challenges they present.^78^ This approach ensures that treatment strategies not only remove contaminants but also mitigate their broader ecological consequences (Table 2).

**Table 2:** Sources, ecological impacts, and treatment options of toxic metals

Toxic metals	Main sources	Ecological impacts	Treatment options
Cadmium (Cd)	Mining, industrial waste, fertilizers	Disruption of microbial activity, soil infertility, bioaccumulation in aquatic systems	Phytoremediation, microbial remediation, chemical precipitation
Lead (Pb)	Battery manufacturing, mining, paint	Toxic to microorganisms, neurotoxicity in humans and animals, soil contamination	Adsorption techniques, phytoremediation, soil washing
Nickel (Ni)	Electronic waste, mining, industrial emissions	Soil contamination, toxicity to plants, bioaccumulation in food chains	Phytoremediation, adsorption, advanced oxidation
Indium (In)	Electronics, mining, industrial waste	Accumulation in sediments, disruption of aquatic ecosystems	Sediment remediation, adsorption, microbial techniques
Mercury (Hg)	Coal combustion, mining, waste incineration	Bioaccumulation in aquatic food webs, neurotoxicity in humans and animals	Chemical precipitation, microbial remediation, activated carbon adsorption

## 6 Challenges and future directions

Addressing toxic metal pollution in the soil and sediment is not without challenges. This section highlights some of the key challenges faced in the mitigation of toxic metal pollution and discusses potential future directions. Long-term monitoring is essential to determine whether the pollution can be remedied in time and track changes in toxic metal contamination levels over time.^79^ Developing standardized monitoring protocols and ensuring continued funding and commitment to monitoring initiatives is crucial. Efforts to remediate toxic metal-contaminated sites should prioritize sustainability.^80^ This includes minimizing the environmental footprint of remediation technologies, considering the energy and resource requirements, and promoting practices that align with broader sustainability goals.^81^ Effectively communicating the risks associated with toxic metal pollution to stakeholders, including communities living near contaminated sites, is essential. Transparent and accessible information can foster trust and encourage proactive measures to address the issue.^82^ Environmental contamination is continually evolving, with new emerging contaminants and their associated risks.^83^ Research and monitoring efforts should remain vigilant in identifying and addressing emerging contaminants to protect the environment and public health.

Future research should prioritize several key areas to address the growing challenges of toxic metal contamination and enhance the effectiveness of remediation strategies.

### 6.1 Advancement of composite materials for remediation

The application of composite materials in the removal of toxic metal ions and organic pollutants presents significant potential. Future research should explore innovative designs of composite materials that optimize their microstructure to enhance adsorption capacity, durability, and reusability. This could include the development of hybrid materials that combine organic and inorganic components, such as nanocomposites capable of simultaneously targeting multiple contaminants. Moreover, studies should investigate how these materials behave under real-world environmental conditions, such as fluctuating temperatures, pH variations, and the presence of co-contaminants, to ensure their robustness in practical applications.

### 6.2 Advanced oxidation technologies

Advanced oxidation processes (AOPs), including Fenton, persulfate oxidation, and electrochemical oxidation, are promising methods for degrading persistent organic pollutants and metals. Future research should focus on optimizing these technologies to reduce energy consumption and enhance their selectivity for different contaminants. A deeper understanding of reaction kinetics, degradation mechanisms, and by-product formation is essential to avoid generating secondary pollutants. Additionally, combining AOPs with other methods, such as adsorption or biological remediation, could create more comprehensive multi-stage remediation strategies that leverage the strengths of each technique.^84^

### 6.3 Innovative analytical and monitoring technologies

Detecting toxic metals at trace levels in complex environmental matrices requires highly sensitive and selective analytical tools. Future research should focus on developing and refining biosensors, nanotechnology-based detectors, and real-time monitoring systems. These technologies should enable faster, more accurate detection of contaminants in water, soil, and air, allowing for immediate response to pollution events. Research should also explore how these systems can be integrated with data analytics, machine learning, and predictive modeling to improve the decision-making process in environmental monitoring and remediation.^85^

### 6.4 Genetic engineering and synthetic biology for bioremediation

In the field of bioremediation, genetic engineering and synthetic biology offer transformative possibilities for enhancing the capabilities of microorganisms and plants to remove or neutralize toxic metals. Future research should focus on engineering microbial strains with increased metal tolerance, bioaccumulation capacity, and metabolic efficiency. Additionally, synthetic biology could be used to modify plant species to hyperaccumulate specific metals or to better survive in contaminated environments. Beyond creating genetically engineered organisms, research should also consider their ecological impacts, ensuring that these species do not disrupt local ecosystems or lead to unintended consequences.^86^

### 6.5 Exploring synergistic remediation approaches

Combining different remediation technologies can lead to synergistic effects that improve overall efficiency. For instance, coupling phytoremediation with microbial-assisted processes could enhance metal uptake by plants while simultaneously promoting soil health through microbial activity. Research should investigate how different technologies, such as adsorption, AOPs, bioremediation, and electrochemical methods, can be integrated into holistic remediation strategies that address both organic and inorganic contaminants across various environmental contexts.^87^

### 6.6 Climate change and contaminant behavior

The effects of climate change on the behavior and mobility of toxic metals in ecosystems need further attention. Research should explore how changing precipitation patterns, rising temperatures, and extreme weather events affect the distribution, bioavailability, and toxicity of heavy metals in both terrestrial and aquatic systems. Understanding these dynamics will be crucial for developing adaptive remediation strategies that remain effective under shifting environmental conditions.

### 6.7 Policy, social science, and public engagement

Effective remediation requires not only technical innovation but also supportive regulatory frameworks and public engagement. Future research should integrate environmental engineering with social science to study how regulatory policies can be improved to encourage sustainable remediation practices. This includes assessing the cost-benefit of different remediation technologies within the context of public health and environmental justice. Engaging local communities in the remediation process, particularly those in areas heavily affected by pollution, can improve transparency and ensure that remediation efforts align with social needs and expectations.^88^

### 6.8 Economic feasibility and scalability

The economic aspects of remediation technologies are critical for their widespread adoption. Future research should assess the cost-effectiveness of emerging technologies, considering factors such as installation, operation, maintenance, and long-term monitoring costs. Additionally, scalability is a significant challenge, especially for biological and adsorption methods, which often perform well in laboratory settings but struggle with large-scale deployment. Research should explore ways to scale these technologies for industrial and regional applications while maintaining their effectiveness and minimizing costs.

By addressing these key areas, future research can be crucial in developing more efficient, sustainable, and adaptable remediation strategies. This holistic approach, combining scientific innovation with policy, economic analysis, and public involvement, will be essential for overcoming the increasingly complex challenges posed by environmental contamination.

## 7 Novelty and contribution

This review provides a novel perspective by focusing on emerging toxic metals like lithium, nickel, and indium, which have been less extensively covered in previous reviews. While many existing reviews discuss common toxic metals such as cadmium and lead, this study expands the focus to include metals that are becoming increasingly important due to their role in modern technology, particularly in electronics and renewable energy. Another key contribution is the integration of biological, chemical, and physical treatment technologies within a comprehensive framework, offering a detailed comparison of their effectiveness, cost, sustainability, and environmental impact. This multidimensional approach differs from previous reviews that often focus on a single remediation method, such as phytoremediation or chemical precipitation, without exploring the synergies between methods. Additionally, this review emphasizes the interdisciplinary approaches necessary for future remediation strategies, combining insights from environmental science, engineering, policy, and social science. This holistic perspective is rarely found in the existing literature and highlights the importance of integrating regulatory and economic considerations with technical solutions. By providing a comprehensive overview of both emerging contaminants and cutting-edge remediation technologies, this review offers valuable guidance for researchers and policymakers seeking to address the evolving challenges posed by toxic metal pollution.

## 8 Conclusion

The increasing reliance on toxic metals in modern technology necessitates a comprehensive approach to address the environmental challenges they pose. By integrating insights from plant science, environmental engineering, and policy, we can effectively navigate the complexities of toxic metal pollution. This review sheds light on the current status of toxic metal pollution, elucidating its sources, distribution, ecological impacts, and treatment technologies. To safeguard the ecosystems and human health, future research endeavors should prioritize translating laboratory findings into practical applications, fostering sustainable toxic metal management practices. Addressing toxic metal pollution is imperative for preserving the environment and ensuring the well-being of future generations. Continued research, monitoring, and sustainable remediation efforts are essential steps towards mitigating the impact of toxic metal contamination and creating a healthier and more sustainable environment for all.

## Ethics approval

Written informed consent for publication of this paper was obtained from the Shandong Jianzhu University and all authors.

## Data availability

Data sharing is not applicable to this article as no new data were created or analyzed in this study.

## Author contributions

Yanhao Zhang: writing – original draft. Zhiyuan Shen: writing – review & editing. Wenlu Zhou: writing – review & editing. Chengying Liu: conducting the research and investigation process. Yi Li: development or design of methodology. Botao Ding: management and coordination responsibility for research activity planning and execution. Peng Zhang: conducting the research and investigation process. Xu Zhang: formulation or evolution of overarching research goals and aims. Zhibin Zhang: acquisition of the financial support for the project leading to this publication.

## Conflicts of interest

The authors have no competing interests to declare that are relevant to the content of this article.

## References

[cit1] Wang R., Liu T., Lu C., Zhang Z., Guo P., Jia B., Hao B., Wang Y., Guo W. (2023). Bioorganic fertilizers improve the adaptability and remediation efficiency of Puccinellia distans in multiple heavy metal-contaminated saline soil by regulating the soil microbial community. J. Hazard. Mater..

[cit2] Ghulam S. T., Abushammala H. (2023). Challenges and opportunities in the management of electronic waste and its impact on human health and environment. Sustainability.

[cit3] Mishra S., Ghosh S., van Hullebusch E. D., Singh S., Das A. P. (2023). A critical review on the recovery of base and critical elements from electronic waste-contaminated streams using microbial biotechnology. Appl. Biochem. Biotechnol..

[cit4] Jain M., Kumar D., Chaudhary J., Kumar S., Sharma S., Verma A. S. (2023). Review on E-waste management and its impact on the environment and society. Waste Management Bulletin.

[cit5] Saha L., Kumar V., Tiwari J., Rawat S., Singh J., Bauddh K. (2021). Electronic waste and their leachates impact on human health and environment: global ecological threat and management. Environ. Technol. Innovation.

[cit6] Thakur P., Kumar S. (2022). Evaluation of e-waste status, management strategies, and legislations. Int. J. Environ. Sci. Technol..

[cit7] Chauhan G., Jadhao P. R., Pant K., Nigam K. (2018). Novel technologies and conventional processes for recovery of metals from waste electrical and electronic equipment: challenges & opportunities–a review. J. Environ. Chem. Eng..

[cit8] Manzoor H., Zawawi M. M., Pakhuruddin M., Ng S., Hassan Z. (2021). High conversion and quantum efficiency indium-rich p-InGaN/p-InGaN/n-InGaN solar cell. Phys. B.

[cit9] Aivali S., Beaumont C., Leclerc M. (2023). Conducting polymers: towards printable transparent electrodes. Prog. Polym. Sci..

[cit10] Deng S., Zhang X., Zhu Y., Zhuo R. (2024). Recent advances in phyto-combined remediation of heavy metal pollution in soil. Biotechnol. Adv..

[cit11] Kurniawan T. A., Lo W.-H., Liang X., Goh H. H., Othman M. H. D., Chong K.-K., Mohyuddin A., Kern A. O., Chew K. W. (2023). Heavy metal removal from aqueous solutions using biomaterials and/or functional composites: recent advances and the way forward in wastewater treatment using digitalization. J. Compos. Sci..

[cit12] Amato A., Rocchetti L., Beolchini F. (2017). Environmental impact assessment of different end-of-life LCD management strategies. Waste Manag..

[cit13] Amato A., Rocchetti L., Fonti V., Ruello M. L., Beolchini F. (2016). Secondary indium production from end-of-life liquid crystal displays. Phys. Status Solidi C.

[cit14] Bolan S., Padhye L. P., Mulligan C. N., Alonso E. R., Saint-Fort R., Jasemizad T., Wang C., Zhang T., Rinklebe J., Wang H. (2023). Surfactant-enhanced mobilization of persistent organic pollutants: potential for soil and sediment remediation and unintended consequences. J. Hazard. Mater..

[cit15] Jensen H., Gaw S., Lehto N. J., Hassall L., Robinson B. H. (2018). The mobility and plant uptake of gallium and indium, two emerging contaminants associated with electronic waste and other sources. Chemosphere.

[cit16] Ali H., Khan E., Ilahi I. (2019). Environmental chemistry and ecotoxicology of hazardous heavy metal: environmental persistence, toxicity, and bioaccumulation. J. Chem..

[cit17] Flora S. J., Pachauri V. (2010). Chelation in metal intoxication. Int. J. Environ. Res. Public Health.

[cit18] Hao X., Ouyang W., Gu X., He M., Lin C. (2024). Accelerated export and transportation of heavy metal in watersheds under high geological backgrounds. J. Hazard. Mater..

[cit19] Liu L., Li Y., Gu X., Tulcan R. X. S., Yan L., Lin C., Pan J. (2025). Priority sources identification and risks assessment of heavy metal (loid) s in agricultural soils of a typical antimony mining watershed. J. Environ. Sci..

[cit20] Nawrot N., Wojciechowska E., Rezania S., Walkusz-Miotk J., Pazdro K. (2020). The effects of urban vehicle traffic on heavy metal contamination in road sweeping waste and bottom sediments of retention tanks. Sci. Total Environ..

[cit21] Andersen J. C. Ø., Cropp A., Paradise D. C. (2017). Solubility of indium-tin oxide in simulated lung and gastric fluids: pathways for human intake. Sci. Total Environ..

[cit22] Dagdag O. , Quadri T. W., Haldhar R., Kim S.-C., Daoudi W., Berdimurodov E., Akpan E. D. and Ebenso E. E., An overview of heavy metal pollution and control, Heavy Metal in the Environment: Management Strategies for Global Pollution, 2023, pp. 3–24

[cit23] Aide M. (2009). Three Missouri Alfisols impacted by aeolian deposition of lead-zinc-cadmium-silver-indium bearing mine tailings. Soil Sediment Contam..

[cit24] White S. J. O., Hussain F. A., Hemond H. F., Sacco S. A., Shine J. P., Runkel R. L., Walton-Day K., Kimball B. A. (2017). The precipitation of indium at elevated pH in a stream influenced by acid mine drainage. Sci. Total Environ..

[cit25] Huang Y., Wang M., Liu B., Su S., Sun H., Yang S., Han G. (2024). The Extraction and Separation of Scarce Critical Metals: A Review of Gallium, Indium and Germanium Extraction and Separation from Solid Wastes. Separations.

[cit26] Shan L., Xiaoyi W., Jun L., Yanan Z., Yuanfeng W. (2023). Impact of metallurgy on the environment: based on an illegal mining pollution case in Beijing, China. Int. J. Environ. Sci. Technol..

[cit27] Nassiri O., Rhoujjati A., Moreno-Jimenez E., Hachimi M. L. E. (2023). Environmental and geochemical characteristics of heavy metal in soils around the former mining area of zeïda (high moulouya, Morocco). Water, Air, Soil Pollut..

[cit28] Zeng J., Tabelin C. B., Gao W., Tang L., Luo X., Ke W., Jiang J., Xue S. (2023). Heterogeneous distributions of heavy metals in the soil-groundwater system empowers the knowledge of the pollution migration at a smelting site. Chem. Eng. J..

[cit29] Shammi S. A., Salam A., Khan M. A. H. (2021). Assessment of heavy metal pollution in the agricultural soils, plants, and in the atmospheric particulate matter of a suburban industrial region in Dhaka, Bangladesh. Environ. Monit. Assess..

[cit30] Sharifi S. A., Zaeimdar M., Jozi S. A., Hejazi R. (2023). Effects of Soil, Water and Air Pollution with Heavy Metal Ions Around Lead and Zinc Mining and Processing Factories. Water, Air, Soil Pollut..

[cit31] Zhang X., Li M., Yang H., Li X., Cui Z. (2018). Physiological responses of Suaeda glauca and Arabidopsis thaliana in phytoremediation of heavy metal. J. Environ. Manage..

[cit32] Yang H., Zhang X., Cui D., Zhu Y. G., Zhang Y., Zhang Z. (2024). Mechanism of flavonols on detoxification, migration and transformation of indium in rhizosphere system. Sci. Total Environ..

[cit33] Zheng K., Benedetti M. F., van Hullebusch E. D. (2023). Recovery technologies for indium, gallium, and germanium from end-of-life products (electronic waste)–a review. J. Environ. Manage..

[cit34] Ayodhya D. (2023). Recent progress on detection of bivalent, trivalent, and hexavalent toxic heavy metal ions in water using metallic nanoparticles: a review. Results Chem..

[cit35] Gravel S., Roberge B., Calosso M., Gagné S., Lavoie J., Labrèche F. (2023). Occupational health and safety, metal exposures and multi-exposures health risk in Canadian electronic waste recycling facilities. Waste Manage..

[cit36] Chang H.-F., Yang P.-T., Hashimoto Y., Yeh K.-C., Wang S.-L. (2023). Temporal transformation of indium speciation in rice paddy soils and spatial distribution of indium in rice rhizosphere. Environ. Pollut..

[cit37] Ladenberger A., Demetriades A., Reimann C., Birke M., Sadeghi M., Uhlbäck J., Andersson M., Jonsson E., Team G. P. (2015). GEMAS: indium in agricultural and grazing land soil of Europe-its source and geochemical distribution patterns. J. Geochem. Explor..

[cit38] Xu Z., Yin M., Yang X., Yang Y., Xu X., Li H., Hong M., Qiu G., Feng X., Tan W. (2024). Simulation of vertical migration behaviors of heavy metal in polluted soils from arid regions in northern China under extreme weather. Sci. Total Environ..

[cit39] Cai W., Navarro D. A., Du J., Srivastava P., Cao Z., Ying G., Kookana R. S. (2023). Effect of heavy metal co-contaminants on the sorption of thirteen anionic per-and poly-fluoroalkyl substances (PFAS) in soils. Sci. Total Environ..

[cit40] Chengatt A. P., Sarath N. G., Sebastian D. P., Mohanan N. S., Sindhu E., George S., Puthur J. T. (2023). Chelate assisted phytoextraction for effective rehabilitation of heavy metal (loid) s contaminated lands. Int. J. Phytorem..

[cit41] Tang J., Zhang L., Zhang J., Ren L., Zhou Y., Zheng Y., Luo L., Yang Y., Huang H., Chen A. (2020). Physicochemical features, metal availability and enzyme activity in heavy metal-polluted soil remediated by biochar and compost. Sci. Total Environ..

[cit42] Lou S., Zou Y., Wang H., Zhou F., Liu S., Tu J., Radnaeva L. D., Nikitina E., Fedorova I. V. (2024). Kinetics release of heavy metal Cu from sediment affected by the mimic vegetation under unidirectional flows and regular waves. Estuarine, Coastal Shelf Sci..

[cit43] Souza L. R. R., Pomarolli L. C., da Veiga M. A. M. S. (2020). From classic methodologies to application of nanomaterials for soil remediation: an integrated view of methods for decontamination of toxic metal(oid)s. Environ. Sci. Pollut. Res..

[cit44] Song X., Zhuang W., Cui H., Liu M., Gao T., Li A., Gao Z. (2022). Interactions of microplastics with organic, inorganic and bio-pollutants and the ecotoxicological effects on terrestrial and aquatic organisms. Sci. Total Environ..

[cit45] Sánchez-Castro I., Molina L., Prieto-Fernández M.-Á., Segura A. (2023). Past, present and future trends in the remediation of heavy-metal contaminated soil-Remediation techniques applied in real soil-contamination events. Heliyon.

[cit46] Mishra S. , Bharagava R. N., More N., Yadav A., Zainith S., Mani S. and Chowdhary P., Heavy metal contamination: an alarming threat to environment and human health, Environmental Biotechnology: For Sustainable Future, 2019, pp. 103–125

[cit47] Zhang Q., Wang C. (2020). Natural and human factors affect the distribution of soil heavy metal pollution: a review. Water, Air, Soil Pollut..

[cit48] Qiu X., Qi Z., Ouyang Z., Liu P., Guo X. (2022). Interactions between microplastics and microorganisms in the environment: modes of action and influencing factors. Gondwana Res..

[cit49] Chatelain M. (2023). Endogeic Earthworms Avoid Soil Mimicking Metal Pollution Levels in Urban Parks. Sustainability.

[cit50] Li T., Wang S., Yu Y., Zong M., Duan C. (2024). Soil microbial communities' contributions to soil ecosystem multifunctionality in the natural restoration of abandoned metal mines. J. Environ. Manage..

[cit51] Labudda M., Dziurka K., Fidler J., Gietler M., Rybarczyk-Płońska A., Nykiel M., Prabucka B., Morkunas I., Muszyńska E. (2022). The alleviation of metal stress nuisance for plants-a review of promising solutions in the face of environmental challenges. Plants.

[cit52] Rahman S. U., Han J.-C., Zhou Y., Ahmad M., Li B., Wang Y., Huang Y., Yasin G., Ansari M. J., Saeed M. (2024). Adaptation and remediation strategies of mangroves against heavy metal contamination in global coastal ecosystems: a review. J. Cleaner Prod..

[cit53] Kou B., Yuan Y., Zhu X., Ke Y., Wang H., Yu T., Tan W. (2024). Effect of soil organic matter-mediated electron transfer on heavy metal remediation: current status and perspectives. Sci. Total Environ..

[cit54] Lu C., Zhang Z., Guo P., Wang R., Liu T., Luo J., Hao B., Wang Y., Guo W. (2023). Synergistic mechanisms of bioorganic fertilizer and AMF driving rhizosphere bacterial community to improve phytoremediation efficiency of multiple HMs-contaminated saline soil. Sci. Total Environ..

[cit55] Tiwari S., Lata C. (2018). heavy metal stress, signaling, and tolerance due to plant-associated microbes: an overview. Front. Plant Sci..

[cit56] Shaffique S., Hussain S., Kang S.-M., Imran M., Kwon E.-H., Khan M. A., Lee I.-J. (2023). Recent progress on the microbial mitigation of heavy metal stress in soybean: overview and implications. Front. Plant Sci..

[cit57] Ali S., Tyagi A., Mushtaq M., Al-Mahmoudi H., Bae H. (2022). Harnessing plant microbiome for mitigating arsenic toxicity in sustainable agriculture. Environ. Pollut..

[cit58] Isinkaralar O., Isinkaralar K., Bayraktar E. P. (2023). Monitoring the spatial distribution pattern according to urban land use and health risk assessment on potential toxic metal contamination via street dust in Ankara, Türkiye. Environ. Monit. Assess..

[cit59] Sharma M., Sharma S., Paavan, Gupta M., Goyal S., Talukder D., Akhtar M. S., Kumar R., Umar A., Alkhanjaf A. A. M., Baskoutas S. (2024). Mechanisms of microbial resistance against cadmium–a review. J. Environ. Health Sci. Eng..

[cit60] Cheah B. H., Liao P.-C., Lo J.-C., Wang Y.-T., Tang I.-C., Yeh K.-C., Lee D.-Y., Lin Y.-F. (2022). Insight into the mechanism of indium toxicity in rice. J. Hazard. Mater..

[cit61] Rahman Z., Singh V. P. (2019). The relative impact of toxic heavy metal(THMs)(arsenic (As), cadmium (Cd), chromium (Cr)(VI), mercury (Hg), and lead (Pb)) on the total environment: an overview. Environ. Monit. Assess..

[cit62] Zhang H., Mao Z., Huang K., Wang X., Cheng L., Zeng L., Zhou Y., Jing T. (2019). Multiple exposure pathways and health risk assessment of heavy metals (loid) s for children living in fourth-tier cities in Hubei Province. Environ. Int..

[cit63] Jiang A., Gong L., Ding H., Wang M. (2021). Cancer mortality and long-term environmental exposure of cadmium in contaminated community based on a third retrospective cause of death investigation of residents living in the Guangdong province from 2004 to 2005. Biol. Trace Elem. Res..

[cit64] Pan F., Yu Y., Yu L., Lin H., Wang Y., Zhang L., Pan D., Zhu R. (2020). Quantitative assessment on soil concentration of heavy metal–contaminated soil with various sample pretreatment techniques and detection methods. Environ. Monit. Assess..

[cit65] Xiao M., Qian L., Yang B., Zeng G., Ren S. (2024). Risk assessment of heavy metal in agricultural soil based on the coupling model of Monte Carlo simulation-triangular fuzzy number. Environ. Geochem. Health.

[cit66] Benalia M. C., Youcef L., Bouaziz M. G., Achour S., Menasra H. (2022). Removal of heavy metal from industrial wastewater by chemical precipitation: mechanisms and sludge characterization. Arabian J. Sci. Eng..

[cit67] Bayuo J., Rwiza M. J., Sillanpää M., Mtei K. M. (2023). Removal of heavy metals from binary and multicomponent adsorption systems using various adsorbents–a systematic review. RSC Adv..

[cit68] Kumar D., Malik S., Rani R., Kumar R., Duhan J. S. (2023). Behavior, risk, and bioremediation potential of heavy metals/metalloids in the soil system. Rendiconti Lincei. Sci. Fis. Nat..

[cit69] Kumar A., Song H.-W., Mishra S., Zhang W., Zhang Y.-L., Zhang Q.-R., Yu Z.-G. (2023). Application of microbial-induced carbonate precipitation (MICP) techniques to remove heavy metal in the natural environment: a critical review. Chemosphere.

[cit70] Wang Z., Zhang H., Xiong Y., Zhang L., Cui J., Li G., Du C., Wen K. (2025). Remediation mechanism of high concentrations of multiple heavy metals in contaminated soil by Sedum alfredii and native microorganisms. J. Environ. Sci..

[cit71] Wu C., Li F., Yi S., Ge F. (2021). Genetically engineered microbial remediation of soils co-contaminated by heavy metals and polycyclic aromatic hydrocarbons: advances and ecological risk assessment. J. Environ. Manage..

[cit72] Barathi S., Lee J., Venkatesan R., Vetcher A. A. (2023). Current Status of Biotechnological Approaches to Enhance the Phytoremediation of heavy metals in India-A Review. Plants.

[cit73] Liu L., Li W., Song W., Guo M. (2018). Remediation techniques for heavy metal-contaminated soils: principles and applicability. Sci. Total Environ..

[cit74] Srivastava A., Gupta B., Majumder A., Gupta A. K., Nimbhorkar S. K. (2021). A comprehensive review on the synthesis, performance, modifications, and regeneration of activated carbon for the adsorptive removal of various water pollutants. J. Environ. Chem. Eng..

[cit75] Saxena G., Purchase D., Mulla S. I., Saratale G. D., Bharagava R. N. (2020). Phytoremediation of heavy metal-contaminated sites: eco-environmental concerns, field studies, sustainability issues, and future prospects. Rev. Environ. Contam. Toxicol..

[cit76] Han Y., Gu X. (2023). Enrichment, contamination, ecological and health risks of heavy metal in agricultural soils of an industrial city, northwestern China. J. Trace Elem. Min..

[cit77] Yang H., Feng Q., Zhu J., Liu G., Dai Y., Zhou Q., Xia S., Wu Z., Zhang Y. (2024). Towards sustainable futures: a review of sediment remediation and resource valorization techniques. J. Cleaner Prod..

[cit78] Zamora-Ledezma C., Negrete-Bolagay D., Figueroa F., Zamora-Ledezma E., Ni M., Alexis F., Guerrero V. H. (2021). heavy metal water pollution: a fresh look about hazards, novel and conventional remediation methods. Environ. Technol. Innovation.

[cit79] Zhang X., Yang H., Cui Z. (2018). Evaluation and analysis of soil migration and distribution characteristics of heavy metal in iron tailings. J. Clean. Prod..

[cit80] Chenavaz R. Y., Dimitrov S., Rustico E. (2023). Green Veblen effect: Sustainability in pollution management. J. Cleaner Prod..

[cit81] Song Y., Pan S., Jin Y., O'Connor D., Nathanail P., Bardos P., Kang Y., Zuo X., Zhang H., Hou D. (2024). Comparative life-cycle sustainability assessment of centralized and decentralized remediation strategies at the city level. Sci. Total Environ..

[cit82] Berti Suman A., Burnette A. (2023). Exploring the Role of Civic Monitoring of Coal Ash Pollution:(Re) gaining Agency by Crowdsourcing Environmental Information. Law Ethics Hum. Right..

[cit83] Khan S., Naushad M., Govarthanan M., Iqbal J., Alfadul S. M. (2022). Emerging contaminants of high concern for the environment: current trends and future research. Environ. Res..

[cit84] Gao Y.-q., Zeng Q., Li K.-x., Chen J.-x., Deng X.-j., Wu T., Li C. (2024). UV-LED cocatalytic Fe3+/peroxymonosulfate process for the degradation of sulfamethoxypyridazine: performance, mechanism and DBP formation during postchlorination. J. Water Proc. Eng..

[cit85] Yadav N., Garg V. K., Chhillar A. K., Rana J. S. (2021). Detection and remediation of pollutants to maintain ecosustainability employing nanotechnology: a review. Chemosphere.

[cit86] Basu S., Rabara R. C., Negi S., Shukla P. (2018). Engineering PGPMOs through gene editing and systems biology: a solution for phytoremediation?. Trends Biotechnol..

[cit87] Mitsova D. (2021). Integrative interdisciplinary approaches to critical infrastructure interdependency analysis. Risk Anal..

[cit88] Selin N. E., Giang A., Clark W. C. (2024). Showcasing advances and building community in modeling for sustainability. Proc. Natl. Acad. Sci. U. S. A..

